# Decreased Concentration of Fibroblast Growth Factor 23 (FGF-23) as a Result of Supplementation with Selenium and Coenzyme Q_10_ in an Elderly Swedish Population: A Sub-Analysis

**DOI:** 10.3390/cells11030509

**Published:** 2022-02-01

**Authors:** Urban Alehagen, Jan Aaseth, Anders Larsson, Jan Alexander

**Affiliations:** 1 Division of Cardiovascular Medicine, Department of Health, Medicine and Caring Sciences, Linköping University, 81 85 Linköping, Sweden; 2 Research Department, Innlandet Hospital Trust, 2381 Brumunddal, Norway; jaol-aas@online.no; 3 Department of Medical Sciences, Uppsala University, 751 85 Uppsala, Sweden; anders.larsson@medsci.uu.se; 4 Norwegian Institute of Public Health, 0403 Oslo, Norway; jan.alexander@fhi.no

**Keywords:** FGF-23, intervention, elderly, selenium, coenzyme Q_10_

## Abstract

There is a reduced intake of selenium in many countries due to low levels of selenium in the soil. This results in an increased cardiovascular risk. Fibroblast growth factor 23 (FGF-23) is active mainly in the metabolism of vitamin D and phosphorus. However, there are indications that FGF-23 may also provide information both on cardiovascular function and prognosis. The aim of the study was to evaluate the effect of supplementation with selenium and coenzyme Q_10_ on the FGF-23 concentration in an elderly population with low concentrations of both selenium and coenzyme Q_10_ and in which the supplementation improved cardiac function and mortality. In a randomised double-blind placebo-controlled trial, FGF-23 was measured in 219 individuals at the start and after 48 months. Selenium yeast (200 µg/day) and coenzyme Q_10_ (200 mg/day) (*n* = 118) or placebo (*n* = 101) were given as a dietary supplement. The intervention time was 48 months. *t*-Tests, repeated measures of variance, and ANCOVA analyses were used to evaluate the differences in FGF-23 concentration. Following supplementation with selenium and coenzyme Q_10_, a significantly lower level of FGF-23 could be seen (*p* = 0.01). Applying 10 years of follow-up, those who later died a cardiovascular death had a significantly higher FGF-23 concentration after 48 months compared with those who survived (*p* = 0.036), and a significantly lower FGF-23 concentration could be seen in those with a normal renal function compared to those with an impaired renal function (*p* = 0.027). Supplementation with selenium and coenzyme Q_10_ to an elderly community-living population low in both substances prevented an increase of FGF-23 and also provided a reduced cardiovascular risk.

## 1. Introduction

Fibroblast growth factor 23 (FGF-23) is a hormone with a molecular weight of 30 kDa and is secreted from the osteocytes and, to a certain extent, from the osteoblasts into systemic circulation where it acts, among other things, on fibroblast growth factor receptors (FGFR1-4) in the kidney, heart, intestine, and parathyroid gland. Some of the main functions of FGF-23 are regulation of the renal vitamin D metabolism and regulation of the phosphorous metabolism [1]. In the kidneys, FGF-23 suppresses proximal tubular phosphate reabsorption, thereby increasing phosphorus excretion, and downregulates formation of 1,25-dihydroxyvitamin D_3_ [2]. In many kidney diseases, the phosphorus excretion declines, and as compensation, the level of FGF-23 increases [3]. As the estimated glomerular filtration rate decreases, a corresponding increase in FGF-23 can be seen [4], and levels of FGF-23 have appeared to predict risk of death in patients with chronic kidney disease [5]. However, there are also reports indicating an association between FGF-23 and cardiovascular (CV) mortality even in the absence of kidney disease [6,7]. Interestingly, experimental data show that FGF-23 may, via specific myocardial FGF receptor activation, act as a mediator for cardiac hypertrophy, cardiac fibrosis, and dysfunction [8].

In accordance with these observations, Reindl et al. reported an association between FGF-23 and left ventricular remodelling of the heart after an ST-elevation myocardial infarction [9]. Chen et al. reported an association between atrial fibrillation and FGF-23 [10], and there are reported associations between FGF-23 and heart failure with preserved or reduced ejection fraction [11,12,13]. There are also reports in the literature that one of the mechanisms where FGF-23 might be involved is general inflammation, which is central in several cardiovascular endpoints [14].

Selenium is an essential trace element, and there are 25 genes encoding human selenoproteins. It is needed in adequate amounts for all human cells to obtain normal cellular functions [15,16]. However, the dietary intake of selenium is low in Europe and in many other areas in the world because of low selenium concentrations in the soil. The mean estimated intake in many European countries is generally < 50 μg/day [17]. To obtain an optimal expression of selenoproteins, however, the required intake of selenium is at least 75 μg/day for an adult Caucasian population [18]. To obtain an optimal expression of one of the important selenoproteins in plasma, selenoprotein P, a daily intake of 100–150 µg/day of selenium is required [19]. Many of the selenoproteins are expressed in the heart and have important functions in redox regulation and protection against oxidative stress. Selenoproteins residing in the endoplasmic reticulum and participating in calcium regulation and protection against misfolded proteins may be of particular importance in the heart [20]. In conditions with inflammation and increased oxidative stress, the need for selenium is increased [21]. Therefore, the intake is lower than required in many parts of the world [15]. Our group has recently reported increased CV mortality in healthy, elderly community-living persons in Sweden due to the low intake of selenium [22].

Coenzyme Q_10_ is an essential substance for human cells. It is a lipid-soluble antioxidant, and it is also part of the mitochondrial respiratory chain. With increasing age, the endogenous production of coenzyme Q_10_ declines. At the age of 80, the myocardial production of coenzyme Q_10_ is about half its amount at 20 years of age [23,24].

In the process of reducing ubiquinone, the oxidised form of Q_10_, into the active form of Q_10_, ubiquinol, the cell needs the selenoenzyme thioreductase1. The human cell, therefore, needs both adequate levels of selenium for an optimal production of the 25 selenoproteins as well as supplementation of coenzyme Q_10_, as the endogenous production decreases with age [23,24]. An insufficiency in selenium could therefore result in decreased concentrations of active coenzyme Q_10_ in the cell [25,26].

Our group has previously reported reduced synthesis of the N-terminal fragment of B-type natriuretic peptide, increased cardiac systolic function, and reduced CV mortality as a result of the supplementation of both selenium and coenzyme Q_10_ in an elderly, community-living “healthy” population in a randomised clinical trial [27]. In addition, we have also reported effects on several biomarkers for inflammation in this population. Thus, the levels of sP-selectin, CRP, osteopontin, osteoprotegerin, soluble tumour necrosis factor receptor 1 (TNFr1), and soluble tumour necrosis factor receptor 2 (TNFr2) were significantly lowered in those receiving active treatment as compared with those in the placebo group [28,29]. The treatment also significantly improved endothelial function as indicated by the levels of the biomarkers plasminogen activator inhibitor 1 and the von Willebrand factor [30].

Apart from a small study reported by Kuklinski et al. on 61 patients with myocardial infarction [31], we have not found any other report in the literature where combined supplementation with selenium and coenzyme Q_10_ has been used. Therefore, the presented results are novel and interesting.

The aim of the present sub-study was to investigate a possible influence of supplementation for four years with selenium and coenzyme Q_10_ on the level of FGF-23 and the potential relation with cardiovascular mortality during 10 years of follow-up in an elderly Swedish population.

## 2. Materials and Methods

### 2.1. Subjects

In a municipality in the south-east of Sweden, all individuals in the age range of 70–88 years were invited to participate in a project where the participants were offered a dietary supplementation consisting of selenium and coenzyme Q_10_ or placebo for four years. Of the 675 individuals invited to participate, 443 accepted. Blood samples were drawn every sixth month [27]. The selenium concentration before intervention was analysed, and the result (mean level 67 μg/L (SD 16.8) (equivalent to an estimated daily intake of 35 μg/day) shows a level well below the selenium concentration considered necessary for optimal physiological supply (≥100 μg/L). Participants were divided into the two groups. In the active treatment group, mean: 66.6 μg/L, SD 15.9; in the placebo group, mean 67.4 μg/L, SD 17.2. In a subgroup consisting of 98 individuals, the concentration was also measured after 48 months, indicating a stable level in the placebo group and a higher level in those who received active treatment, as expected (active treatment group: mean 210.3 μg/L, SD 59.4; placebo group: mean 71.5 μg/L, SD 24.9) [22,32].

In the present sub-analysis of FGF-23 in the intervention project population, only those still alive after 48 months and who agreed to participate in this sub-analysis were included, and thus, the study population consisted of 219 individuals. Of those, 118 individuals were on active treatment, and 101 individuals were on placebo.

The participants received 200 mg/day of coenzyme Q_10_ capsules (Bio-Quinon 100 mg B.I.D, Pharma Nord, Vejle, Denmark) and 200 µg/day of organic selenium yeast tablets (SelenoPrecise 100 µg B.I.D, Pharma Nord, Vejle, Denmark) or placebo over 48 months. After the intervention period, the supplementation was finished. The supplementation was taken in addition to any regular medication. All study medications (active drug and placebo) not consumed were returned and counted. One of three experienced cardiologists examined all the study participants on inclusion. At inclusion, a new clinical history was taken, and a clinical examination was performed, blood pressure was measured, and assessment of New York Heart Association functional class (NYHA class), electrocardiogram (ECG), and Doppler-echocardiography were performed. Echocardiographic examinations were performed with the participant in the left lateral position. The ejection fraction (EF) readings were categorised into four classes: 30%, 40%, and 50% [33,34]. Normal systolic function was defined as EF ≥ 50%, while severely impaired systolic function was defined as EF *<* 30%. Only the systolic function was evaluated. The inclusion started in January 2003 and finished in February 2010.

The exclusion criteria for the main project were recent myocardial infarction (within four weeks); planned cardiovascular operative procedure within four weeks; hesitation concerning whether the candidate could decide for him/herself to participate in the study or not or doubt about whether he/she understood the consequences of participation; serious disease that substantially reduced survival or when it was not expected that the participant could cooperate for the full four-year period; other factors making participation unreasonable or drug/alcohol abuse [27]. CV mortality was registered for all study participants for a follow-up period of 10 years. Information regarding mortality was obtained from the National Board of Health and Welfare in Sweden, which registers all deaths of Swedish citizens based on death certificates or autopsy reports. All patients obtained written informed consent.

CV mortality was defined as mortality due to myocardial infarctions, cerebrovascular lesions, fatal cardiac arrhythmias, heart failure, and aortic aneurysms.

The result of the main study was that the actively treated group showed a significantly increased cardiac systolic function, a reduced concentration of the cardiac peptide N-terminal fragment of B-type natriuretic peptide (NT-proBNP), and significantly reduced cardiovascular mortality [27]. As the result of the main study was surprising, several sub-studies were performed.

### 2.2. Biochemical Analyses

All blood samples were collected at the start of the study and after 48 months and were drawn with the participants resting and in a supine position. Pre-chilled, EDTA vials for plasma were used. The vials were centrifuged at 3000× *g*, +4 °C and were then frozen at −70 °C. No sample was thawed more than once.

### 2.3. Determination of FGF-23

Human FGF-23 was analysed by a commercial sandwich ELISA (DY2604-05, R&D Systems, Minneapolis, MN, USA), in which a monoclonal antibody specific for FGF-23 was coated onto microtitre plates. Standards and samples were pipetted into the wells, and the peptide was bound to the immobilized antibodies. After washing, a biotinylated anti-FGF-23 antibody was added. After incubation and washing, a streptavidine-HRP conjugate was added to the wells. After incubation and washing, a substrate solution was added. The development was stopped, and the absorbance was measured in a SpectraMax 250 (Molecular Devices, Sunnyvale, CA, USA). The concentrations in the samples were determined by comparing the optical density of the sample with the standard curve. The assays were calibrated against highly purified recombinant human FGF-23. Measurements were performed without knowledge of the clinical diagnoses. The samples were analysed during three consecutive days, and the total coefficient of variation during the assay period was approximately 6%.

### 2.4. Statistical Methods

Descriptive data are presented as percentages or mean ± standard deviation (SD). A Student’s unpaired two-sided *t*-test was used for continuous variables, and the chi-square test was used for analysis of one discrete variable. As the FGF-23 concentration differed considerably between the participants at inclusion, we chose to calculate the difference in FGF-23 concentration between inclusion and after 48 months in each individual (δFGF-23 = FGF-23_incl._ − FGF-23_48m_). The calculations were performed on this difference, as it better reflects each individual change. Repeated measures of variance were used in order to obtain better information on the individual changes in the concentration of the biomarker analysed compared to group mean values.

Analysis of covariance (ANCOVA) evaluation was performed on both log_10_ transformed and non-transformed data, with no significant difference in the results.

In the ANCOVA evaluation, the FGF-23 concentration after 48 months was used as a dependent variable. In the model, adjustments were made for C-reactive protein, FGF-23 at inclusion, N-terminal fragment of proBNP, smoking, hypertension, diabetes, NYHA class III, Hb < 120 g/L, male gender, and supplementation with selenium and coenzyme Q_10_. *p*-Values < 0.05 were considered significant based on a two-sided evaluation. All data were analysed using standard software (Statistica v. 13.2, Dell Inc, Tulsa, OK, USA).

## 3. Results

The baseline characteristics for the active treatment and the placebo groups are presented in Table 1. The two groups were well balanced as regards the covariates analysed.

At baseline (Table 1), 48 out of 219 (22%) participants had diabetes, 153 out of 219 (70%) had hypertension, 38 out of 219 (17%) had ischaemic heart disease, and 11 out of 219 (5%) had impaired systolic cardiac function, defined as an EF of less than 40%. The population evaluated could be considered as representative of an elderly Swedish population.

### 3.1. FGF-23 and Atrial Fibrillation

We observed a higher concentration of FGF-23 in the group with atrial fibrillation compared with those having sinus rhythm (atrial fibrillation: 7416 pg/mL; sinus rhythm: 2919 pg/mL; *p* = 0.049).

### 3.2. FGF-23 and 10-Year Mortality

We then compared the group suffering CV mortality within 10 years after the start of the intervention with the non-CV mortality group. For the first group, the concentration of FGF-23 increased by 3527 pg/mL, whereas for the non-CV mortality group, it decreased by 3737 pg/mL (*p* = 0.036).

### 3.3. FGF-23 and Renal Function

The literature also indicates that there is an association between renal function and FGF-23 concentration [35]. We therefore stratified the population in quartiles according to kidney function using the eGFR algorithm of the Chronic Kidney Disease Epidemiology Collaboration (CKD Epi Crea) [36]. We found that in the first quartile (Q1) (<53 mL/min/1.73 m^2^), the FGF-23 decreased between inclusion and after 48 months by 4519 pg/mL, while in Q4 (>78 mL/min/1.73 m^2^), it increased by1300 pg/mL: *p* = 0.027.

### 3.4. Effect of Supplementation on the Concentration of FGF-23

**Figure 1:**
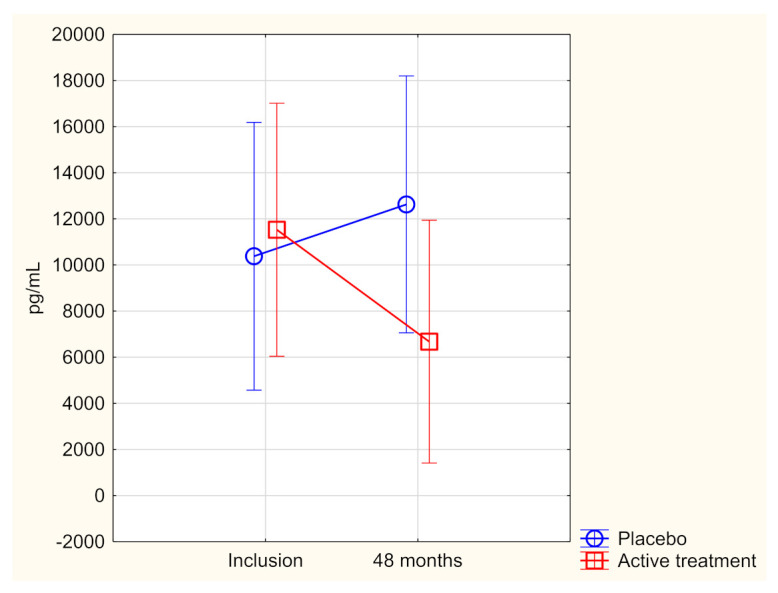
Concentration of FGF-23 at the start of the project and after 48 months in the selenium and coenzyme Q_10_ treatment group compared to the placebo group in the study population.

At inclusion, we found no significant difference in concentration between the active treatment group and the placebo group (active treatment group: 10,834 pg/mL vs. placebo: 10,339 pg/mL; *p* = 0.87). Thereafter, the difference in concentration of FGF-23 between inclusion and after 48 months in those who received supplementation was compared with the difference in those on placebo. A significantly lower value of FGF-23 could be seen in the active treatment group, while in the placebo group, the value increased (difference in FGF-23 between inclusion and after 48 months: active treatment: 5240 pg/mL vs. placebo: −834 pg/mL; *p* = 0.021). In addition, to validate the obtained results, following the individual change in every participant, we applied the repeated measures of variance methodology (Figure 1). A significantly lower level of FGF-23 could be seen in the active treatment group after 48 months (F = 6.60; *p* = 0.011) in comparison with the placebo group.

Evaluation performed by use of repeated measures of variance methodology

Current effect: (F(1,193)= 6.60; *p*= 0.011).

Vertical bars denote 0.95 confidence intervals.

Blue curve: placebo; red curve: active treatment group.

Furthermore, as a third step of validation, the effect of supplementation in relation to other covariates was analysed, and an ANCOVA evaluation was performed (Table 2). This analysis showed that the intervention did have a significant effect on the concentration of FGF-23 even when adjusting for several other clinically important covariates.

## 4. Discussion

In the present study of an elderly, community-living population in Sweden supplemented with selenium and coenzyme Q_10_, we presented the effect of supplementation with selenium and coenzyme Q_10_ on the concentration of FGF-23. The study disclosed a significantly decreased concentration of FGF-23 after the intervention with selenium and coenzyme Q_10_.

We also found a relationship between the circulating level of the biomarker FGF-23 and atrial fibrillation. The latter association is in accordance with previous reports [10]. By applying an observation period of 10 years, we confirmed a previously observed association between the concentration of FGF-23 and CV mortality [6,37]. However, as the increased cardiovascular risk could be explained by other well-known cardiovascular risk factors, such as increased myocardial wall tension, we included the cardiac peptide NT-proBNP as a covariate in the ANCOVA analysis. We found effects on FGF-23 following supplementation of selenium and coenzyme Q_10_ independent of NT-proBNP. This concurs with what has previously been reported in the literature [38].

As FGF-23 is closely interrelated to the renal function [39], it is not surprising that we also observed an association between renal function and the level of FGF-23. Our group has previously reported increased or conserved renal function in elderly individuals low in selenium and Q_10_ upon supplementation with selenium and Q_10_ [40]. As several reports point to the fact that there is an intimate relationship between the level of FGF-23 and renal function, the decrease of FGF-23 could simply be a result of the renal effects of the supplementation. However, another denominator explaining the previous and present findings could be systemic inflammation in the elderly subjects. In a population with a selenium intake that is below levels needed for optimal cellular function, there is an increased level of inflammation that apparently can be reduced by supplementation of selenium and coenzyme Q_10_ [28,29]. Thus, one of the mechanisms behind the reduced FGF-23 levels following the supplementation could be its anti-inflammatory effects. Relevant here are the previous studies showing that pro-inflammatory cytokines, such as IGF-1, TNF-α, and interleukin-6, induce increased FGF-23 synthesis [41,42]. It is also known that there is a close connection between general inflammation and development of cardiovascular disease [28,29,43].

The exact mechanism of the influence of FGF-23 on the cardiovascular system is currently unclear, but it has been shown that many of the effects are mediated by cardiovascular FGF receptors that are regulated by interaction with the protein α-klotho [44]. It is mainly FGF receptor 4 that interacts with the cardiomyocytes [45]. However, Han et al. reported that cardiac hypertrophy, when induced by high levels of FGF-23, could be attenuated by the administration of soluble klotho protein in a mouse model [46], whereas Pastor-Arroyo reported the absence of ventricular hypertrophy in a mouse model without kidney disease [45]. It is known that pro-inflammatory cytokines can induce FGF-23 [47]. Taking all these facts together, there is an intricate relationship between FGF-23 and inflammation, which could be a starting point for many pathological mechanisms also in the cardiovascular system.

However, there are also indications that FGF-23 influences levels of active vitamin D, which in turn influences the cardiovascular risk [48]. In the present project, the vitamin D metabolites have not been determined.

Based on the literature, we argue that our study population, with a low serum selenium concentration that is well below the recommended level [49], is representative of an elderly Swedish population.

In conclusion, the effect of the supplementation with selenium and coenzyme Q_10_ could, besides an impact on renal function [3] be explained by the previously reported decrease in inflammatory activity in the studied elderly populations as a result of the supplementation [28] although it should be noted that even if adjusted for biomarkers of inflammation as covariates, an independent reduction in the level of FGF-23 appeared to persist following the supplementation with selenium and coenzyme Q_10_. Altogether, we consider that the reported results are important to better understand the mechanisms behind the positive cardiovascular findings resulting from supplementation with selenium and coenzyme Q10 in a population low in both of these substances.

### Limitations

The population analysed in this sub-study was of relatively small size. Therefore, we applied a two-step validation process, and from these evaluations, we argue that the results are correct. Even though the size of the study population was small, we regard the results as hypothesis-generating and interesting from a scientific point of view.

The age stratum of the included participants was relatively narrow, so it is not possible to extrapolate the results to other age groups with certainty.

Finally, as the evaluated population consisted of Caucasians who were low in selenium and coenzyme Q_10_, it is not necessarily true that the obtained results could be extrapolated to another selenium-replete population.

## 5. Conclusions

Fibroblast growth factor 23 is a hormone, and its main function is to influence the homeostasis of phosphorus and vitamin D. However, research has indicated it also has an important relationship with cardiovascular function and risk. In this sub-study, we evaluated the effect of supplementation with selenium and coenzyme Q_10_ on FGF-23. We can now report a significantly lower concentration of FGF-23 following an intervention for 48 months. Besides the positive effect of supplementation on renal function in those low in selenium and Q10, a possible relation to inflammation has been discussed as an explanation for the difference in 10-year mortality. The results should be regarded as hypothesis-generating, and it is hoped they will stimulate more research within the same area.

## Figures and Tables

**Table 1:** Baseline characteristics of the study population receiving dietary supplementation of selenium and coenzyme Q_10_ or placebo for a period of four years.

	Active Treatment Group N = 118	Placebo Group N = 101	*p*-Value
Age years, mean (SD)	76.2 (3.1)	76.3 (3.1)	0.74
Gender, Males/Females	58/60	43/58	
**History**			
Diabetes, n (%)	20 (16.9)	18 (17.8)	0.87
Smoking, n (%)	8 (6.8)	9 (8.9)	0.56
Hypertension, n (%)	81 (68.6)	72 (71.3)	0.67
IHD, n (%)	22 (18.6)	16 (15.8)	0.59
Atrial Fibrillation, n (%)	8 (6.8)	7 (6.9)	0.96
NYHA class I, n (%)	71 (60.2)	58 (57.4)	0.68
NYHA class II, n (%)	29 (24.6)	30 (29.7)	0.39
NYHA class III, n (%)	18 (15.3)	12 (11.9)	0.47
NYHA class IV, n (%)	0	0	
Unclassified, n	0	1	
**Medications**			
ACEI/ARB, n (%)	19 (16.1)	21 (20.8)	0.37
Beta blockers, n (%)	44 (37.3)	33 (32.7)	0.48
Diuretics, n (%)	39 (33.1)	33 (32.7)	0.95
Statins, n (%)	27 (22.9)	17 (16.8)	0.20
			
**Examinations**			
EF < 40%, n (%)	7 (5.9)	4 (4.0)	0.51
CV mortality, n (%)	16 (13.6)	24 (23.8)	0.05
Selenium conc. pre-intervention mean, µg/L (SD)	66.6 (15.9)	67.3 (17.2)	0.56

Abbreviations: ACEI, ACE-inhibitors; ARB, angiotension receptor blockers; EF, ejection fraction; IHD, ischaemic heart disease; NYHA, New York Heart Association functional class; SD, standard deviation. Note: Values are given by means ± SDs or frequency (percent). A Student’s unpaired two-sided *t*-test was used for continuous variables, and the chi-square test was used for analysis of one discrete variable.

**Table 2:** Analysis of covariance using FGF-23 after 48 months as a dependent variable.

Effects	Sum of Squares	F	*p*
**Intercept**	5.21 × 10^8^	2.62	0.11
**Age**	6.1 × 10^8^	3.06	0.08
**HsCRP**	1.3 × 10^8^	0.66	0.42
**FGF-23 inclusion**	4.5 × 10^10^	225.3	<0.0001
**NT-proBNP**	1.6 × 10^8^	0.80	0.37
**Smoker**	1.3 × 10^7^	0.07	0.80
**Hypertension**	3.0 × 10^8^	1.49	0.23
**Diabetes**	1.1 × 10^9^	5.51	0.02
**NYHA 3**	6.0 × 10^7^	0.28	0.60
**Hb < 120 g/L**	6.5 × 10^7^	0.33	0.57
**Male**	2.4 × 10^7^	0.12	0.73
**s-selenium microgr/L, incl.**	3.7 × 10^7^	0.18	0.67
**Active treatment**	1.07 × 10^9^	5.35	0.02
**Error**	1.6 × 10^10^		

Note: HsCRP, high-sensitivity assay of CRP; IHD, ischaemic heart disease; NT-proBNP, N-terminal fragment of B-type natriuretic peptide; NYHA 3, New York Heart Association functional class 3.

## Data Availability

Under Swedish Law, the authors cannot share the data used in this study and cannot conduct any further research other than that specified in the ethical permissions application. For inquiries about the data, researchers should first contact the owner of the database, the University of Linköping. Please contact the corresponding author with requests for and assistance with data. If the university approves the request, researchers can submit an application to the Regional Ethical Review Board for the specific research question that the researcher wants to examine.

## Abbreviations

ACEI	ACE inhibitors
ANCOVA	Analysis of covariance
ARB	Angiotension receptor blockers
CRP	C-reactive protein
CV	Cardiovascular
EF	Ejection fraction
ECG	Electrocardiogram
Hs-CRP	High-sensitivity analysis of C-reactive protein
IHD	Ischaemic heart disease
NT-proBNP	N-terminal fragment of B-type natriuretic peptide
NYHA class	New York Heart Association functional class
SD	Standard deviation
